# Two-Dimensional Transition Metal Oxide and Hydroxide-Based Hierarchical Architectures for Advanced Supercapacitor Materials

**DOI:** 10.3389/fchem.2020.00390

**Published:** 2020-05-15

**Authors:** Meili Guan, Qiuwan Wang, Xuan Zhang, Jian Bao, Xuezhong Gong, Youwen Liu

**Affiliations:** ^1^Institute for Energy Research, School of Chemistry and Chemical Engineering, Jiangsu University, Zhenjiang, China; ^2^National Center of International Joint Research for Hybrid Materials Technology, National Base of International Science and Technology Cooperation on Hybrid Materials, College of Materials Science and Engineering, Institute of Hybrid Materials, Qingdao University, Qingdao, China; ^3^State Key Laboratory of Material Processing and Die & Mould Technology, School of Materials Science and Engineering, Huazhong University of Science and Technology, Wuhan, China

**Keywords:** two-dimensional nanosheets, transition metal oxide and hydroxide, hierarchical structure, integration, supercapacitor

## Abstract

The supercapacitor has been widely seen as one of the most promising emerging energy storage devices, by which electricity is converted from chemical energy and stored. Two-dimensional (2D) metal oxides/hydroxides (TMOs/TMHs) are revolutionizing the design of high-performance supercapacitors because of their high theoretical specific capacitance, abundance of electrochemically active sites, and feasibility for assembly in hierarchical structures by integrating with graphitic carbon, conductive polymers, and so on. The hierarchical structures achieved can not only overcome the limitations of using a single material but also bring new breakthroughs in performance. In this article, the research progress on 2D TMOs/TMHs and their use in hierarchical structures as supercapacitor materials are reviewed, including the evolution of supercapacitor materials, the configurations of hierarchical structures, the electrical properties regulated, and the existence of advantages and drawbacks. Finally, a perspective covering directions and challenges related to the development of supercapacitor materials is provided.

## Introduction

Global warming and the rapid depletion of fossil fuels are driving the development of sustainable and renewable energy. At present, the renewable energy sources of wind, tidal, geothermal, and solar energy have been researched and utilized (Yuksel and Ozturk, 2017; Cranmer et al., 2018; Mo et al., 2018; Song et al., 2018). However, these energy sources are highly dependent on nature, which is variable and unpredictable. Hence, an energy storage system is essential for efficiently collecting this natural energy, and, meanwhile, allows the energy to be rapidly exported in the form of stable electric energy. In recent years, the supercapacitor is becoming popular as a kind of energy storage device that is not only efficient and practical but also convenient and environmental friendly (Lukatskaya et al., 2016; Salanne et al., 2016; Wang et al., 2017).

The supercapacitor is also known as a Faraday quasi capacitor or electrochemical capacitor. It stores charges through a reversible redox reaction at the interface between electrode materials and electrolyte, unlike a traditional capacitor, it offers higher specific capacity and energy density (Simon and Gogotsi, 2010; Kate et al., 2018). Compared with a secondary battery, the supercapacitor possesses overwhelming advantages in terms of a shorter charging time and a long cycle life and, higher power density (Staaf et al., 2014; Zhao and Sun, 2018). Therefore, the supercapacitor is a kind of energy storage device that is intermediate between a traditional capacitor and a secondary battery that can be regarded as a complementary device between a battery and a traditional capacitor and can meet the needs of human beings for new energy.

The structural configuration of a supercapacitor mainly consists of electrode material, a diaphragm, electrolyte, and collecting fluid (Muzaffar et al., 2019). The energy storage is mainly from the charge transfer process at the interface of electrode and electrolyte, so the electrode material is the key factor in the performance of the supercapacitor. At present, there are three prevailing types of electrode materials under research, namely carbon materials [e.g., graphene (Liu et al., 2010), carbon nanotubes (Shang et al., 2015), activated carbon (Gamby et al., 2001; Jin et al., 2016), and porous carbon (Bu et al., 2017)], conducting polymer materials [e.g., polyaniline (PANI) (Lim et al., 2015; Lyu et al., 2019), polypyrrole (PPy) (Huang et al., 2015), polythiophene (PTh) (Ambade et al., 2017), and poly (3, 4-ethylenedioxythiophene) (PEDOT) (Liu et al., 2008)], and TMOs/TMHs [RuO_2_ (Sugimoto et al., 2003), MnO_2_ (Song et al., 2010; Zhao et al., 2012; Huang et al., 2018), MoO_3_ (Brezesinski et al., 2010; Hanlon et al., 2014), Nb_2_O_5_ (Augustyn et al., 2013), V_2_O_5_ (Qi et al., 2018), Ni(OH)_2_ (Ida et al., 2008; Fu et al., 2014), and Co(OH)_2_ (Ji et al., 2012; Gao et al., 2014)]. In particular, carbon material is the most widely used electrode material due to its diversity and abundance as a resource. The capacitance over a carbon-based supercapacitor electrode is achieved through pure electrosorption of the electrical double-layer; thus, the structure of the electrode material will not collapse with the insertion and withdrawn of electrolyte ions during the charging and discharging processes. This leads to superior cycle stability, extending to tens of thousands or even hundreds of thousands of cycles (Frackowiak and Beguin, 2001; Liu et al., 2014). However, the energy density of carbon-based supercapacitors is low due to the storage mechanism, which rarely meets the needs of practical application. In contrast, conducting polymer electrode materials achieve high charge density by a reversible redox reaction of elements doping/dedoping, thus realizing large electric energy storage (Snook et al., 2011). However, the conducting polymer skeleton is prone to expanding and shrinking in the charging–discharging process, leading to low cycle stability (González et al., 2016). In addition, most of the conducting polymer materials are dense structures with limited interfaces, which restricts the amount of electrode material that is able to fully contact with the electrolyte and results in relatively low power density.

It is noteworthy that TMOs/TMHs exhibit potential for the fabrication of supercapacitor electrodes due to their high theoretic capacity and ultrahigh power density (Yuan et al., 2014; Wang et al., 2015; Zhang H. et al., 2015; Nguyen and Montemor, 2019). The energy storage can be achieved by either electrosorption or reversible redox reactions (Lee et al., 2010). Normally the valance state would change accompany with the charging–discharging process. Hence, for comparison, TMOs/TMHs have exhibited higher powder density and stability than traditional carbon and conducting polymer materials. Although problems arise from their narrow working voltage window, low conductivity, and small reaction area, these can be solved by structural engineering and composition, such as by controlling dimensionality and morphology (Zhu et al., 2014; Najafpour et al., 2015; Xu et al., 2015; Yue and Liang, 2015; Yu et al., 2015).

In this review, we summarize the recent progress in 2D TMOs/TMHs as supercapacitor electrode material. Further, 2D transition metal oxide/hydroxide-based hierarchical structures are covered. The different technical strategies in design and synthesis for optimizing the conductivity, structural stability, surface physical, and chemical properties, and structural morphology of the hierarchical composites are also detailed. A supercapacitor electrode with high specific capacitance, good rate capability, and excellent cycle stability can be obtained by tuning the electrochemical properties of the electrode materials. Finally, a perspective covering directions and challenges related to the development of supercapacitor materials is provided.

## Two-Dimensional Transition Metal Oxides/Hydroxides

Transition metal oxides/hydroxides (TMOs/TMHs) are the most representative active electrochemical pseudocapacitor materials and are widely known for their high theoretical capacitance, abundance in nature, and high energy density (Jiang et al., 2012). However, poor intrinsic conductivity and other shortcomings have limited their performance as supercapacitor materials. Hence, 2D ultrathin TMOs/TMHs have been widely studied and applied in supercapacitors due to their advantages of high specific surface area and improved planar electronic conductivity (Gao et al., 2014; Liu W. et al., 2017; Zhu et al., 2018). Most importantly, the atomic thickness shortens the ion diffusion path and reduces ion diffusion resistance.

So far, there are two main strategies, that is, “top-down” and “bottom-up” methods, for obtaining 2D TMOs/TMHs (Sun et al., 2017; Mei et al., 2018), as shown in Table 1. “Top-down” mainly refers to the chemical or mechanical exfoliation of layered bulk materials, such as by mechanical exfoliation and liquid-phase exfoliation (Coleman et al., 2011; Rui et al., 2013; Zhang Y. Z. et al., 2015; Peng et al., 2017; Zavabeti et al., 2017; Tao et al., 2019). It is reported that MoO_3_, MnO_2_, and RuO_2_ nanosheets, etc., can be made at a large scale by ultrasonic exfoliation in ethanol/water and can be applied to solid-state symmetrical supercapacitors (Dutta et al., 2019). A liquid-phase exfoliation method with the aid of lithium (Li) intercalation and de-intercalation was developed for the preparation of quasi-layered VO_2_ ultrathin nanosheet (Liu et al., 2012). The underlying mechanism is the insertion of Li ions to break the chemical bonds of the layers. Subsequent replacement of Li ions by larger molecules and ultrasonic treatment can result in a dispersed VO_2_ ultrathin nanosheet. The “bottom-up” synthetic protocol starts from appropriate design at the atomic or molecular level by the aid of technologies, such as self-assembly (Sun J. et al., 2014; Hu et al., 2017; Sun et al., 2017), chemical vapor deposition (Lee and Sung, 2012; Liu et al., 2015), directional connection, and topological chemical transformation of layered intermediates (Liu et al., 2016; Wen et al., 2017; Bao et al., 2018), to synthesize a 2D nanostructure. For instance, five atomic layer thickness Co(OH)_2_ ultrathin nanosheet was synthesized by the orientation connection method and was assembled into an asymmetric vertical all-solid-state flexible supercapacitor with excellent specific capacitance and cycle stability (Gao et al., 2014). For non-layered 2D materials, Qiu and Zheng, for the first time, successfully observed the *in-situ* transformation of metal oxides from 3D nanoparticles as intermediate products to 2D oxide nanoflakes by LBNL's *in-situ* liquid-phase transmission electron microscopy technology, revealing the new evolution strategy of 3D to 2D materials at the atomic level, and also paving the way to obtaining ultrathin nanostructures from non-layered materials (Yang et al., 2019).

**Table 1:** Summary and comparison between top-down and bottom-up strategies for the fabrication of 2D TMOs/TMHs and their pros and cons.

**Strategy**	**Method**	**2D TMOs/TMHs**	**References**	**Pros and cons**
Top-down	Mechanical cleavage	MnO_2_	Peng et al., 2017; Dutta et al., 2019	Easy operation; Only applicable for layered structure materials
		MoO_3_	Dutta et al., 2019	
		RuO_2_	Dutta et al., 2019	
		Ti_5_NbO_14_	Zhang et al., 2010	
	Liquid-phase exfoliation	V_2_O_5_	Rui et al., 2013	
		VO_2_	Liu et al., 2012	
		MoO_3_	Zhang Y. Z. et al., 2015	
Bottom-up	Self-assembly	TiO_2_	Sun J. et al., 2014	Applicable for both layered and non-layered structure materials; Can be produced at large scale; Harsh synthetic conditions; Difficult to obtain high-quality 2D crystals.
		ZnO	Sun et al., 2017	
		Co_3_O_4_	Hu et al., 2017	
		WO_3_	Sun J. et al., 2014	
	Chemical vapor deposition	TiO_2_	Lee and Sung, 2012	
		WO_3_	Liu et al., 2015	
	Directional connection	SnO_2_	Wang C. et al., 2012	
		CeO_2_	Yu et al., 2010	
		Co(OH)_2_	Gao et al., 2014	
	Topological chemical transformation	WO_3_	Liu et al., 2016	
		Nb_2_O_5_	Wen et al., 2017	
		ZnCo_2_O_4_	Bao et al., 2018	
		Cobalt nickel oxide	Yang et al., 2019	

## 2D TMOs/TMHs-Based Hierarchical Engineering for Supercapacitor Materials

**Figure 1:**
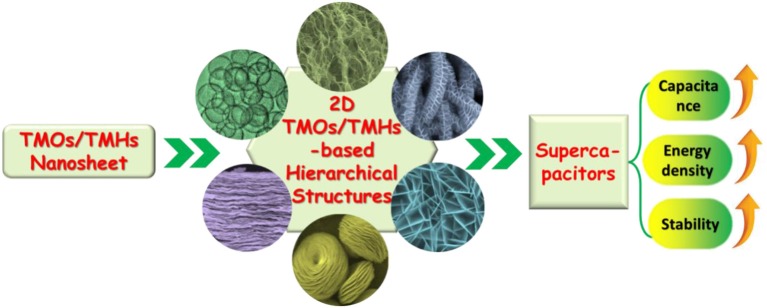
Schematic illustration of the use of TMOs/TMHs nanosheets to form 2D TMOs/TMHs-based hierarchical structures for a supercapacitor with expected performance promotion.

Although great efforts have been put into the development of 2D TMOs/TMHs for supercapacitor electrodes, challenges still exist when using a single electrode material. The drawback lies in low actual capacitance, limited improvement of energy density, and the poor rate capability caused by low conductivity, which have restricted the performance of such supercapacitors in practical applications (Jiang et al., 2012; Mahmood et al., 2019). In order to overcome the limitations of single electrode materials, it is necessary to combine 2D TMOs/TMHs with other low-dimensional nanomaterials to construct hierarchical nanostructures, which can not only optimize the configuration to overcome the agglomeration of 2D nanosheets but also complement and enhance the performance of different electrode materials so as to realize effective improvement of the performance for the supercapacitor device (Figure 1).

## TMOs/TMHs Hierarchical Structures

The engineering of hierarchical structures by hybridizing different TMOs/TMHs is regarded as an efficient strategy to combine the advantages of different materials (Wu et al., 2013; Dinh et al., 2014; Zheng et al., 2016, 2018; Ouyang et al., 2019). The function of this kind of hierarchical structure can be generally summarized as overcoming the drawbacks of the individual components and preventing agglomeration of nanosheets. Thus, capacitance performance is predominantly enhanced because of the expected synergistic effect. Hierarchical structures with different morphologies can be formed through self-assembly (Huang et al., 2014; Feng et al., 2018), layer stacking (Zhu et al., 2012), and heterostructure core-shell engineering (Li et al., 2014; Sun Z. et al., 2014; Ho and Lin, 2019), which also have significant effects on the energy density and stability of the supercapacitors.

**Figure 2:**
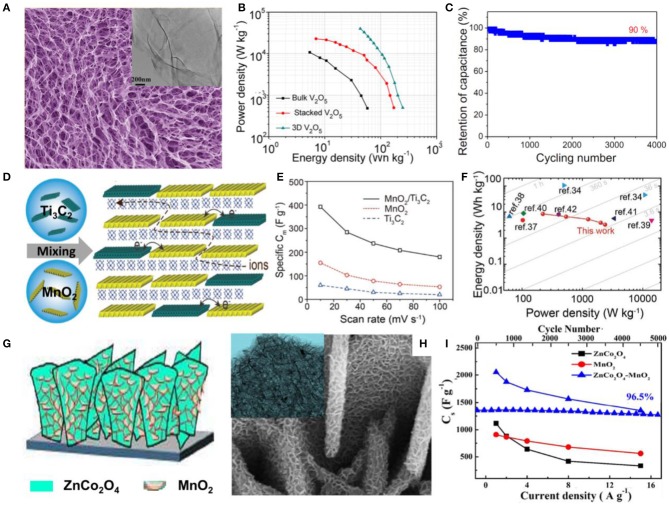
**(A)** FESEM images of the 3D V_2_O_5_ architecture constructed from nanosheets (inset: TEM image of a nanosheet). **(B)** Power density and energy density of three electrodes. **(C)** Cycling performance of the 3D V_2_O_5_ architecture (Zhu et al., 2013). **(D)** Schematic drawing of the fabrication of the MnO_2_/Ti_3_C_2_ hybrid film, with molecular sheets stacked in a randomly interstratified manner. **(E)** Rate capability of the hybrid MnO_2_/Ti_3_C_2_ electrode compared with neat MnO_2_ and Ti_3_C_2_ electrodes. **(F)** Performance comparison of the MnO_2_/Ti_3_C_2_ hybrid with other reported materials on a Ragone plot (Liu W. et al., 2017). **(G)** Schematic of the coating of ZnCo_2_O_4_ nanoflakes with MnO_2_ nanosheets. **(H)** FESEM images and (inset) TEM images of ZnCo_2_O_4_ nanoflakes coated with MnO_2_ nanosheets. **(I)** Variation in C_s_ with respect to current density and cycling performance at 15 A/g for electrodes (Gao et al., 2017).

A 3D V_2_O_5_ architecture was constructed by using ultrathin V_2_O_5_ nanosheets as building blocks with a thickness of 4 nm via a freeze-drying process (Zhu et al., 2013). Due to the benefits of its porous and ultrathin nature, as well as the 3D interpenetrating structure, a high surface area and shortened diffusion length were expected, thus enabling such a supercapacitor electrode to exhibit high capacitance, high energy density, and excellent stability (Figures 2A–C). A flexible film-like supercapacitor was fabricated via a vacuum filtration method by integrating MnO_2_ and Ti_3_C_2_ nanoflakes as the electrode, as shown in Figure 2D (Liu Y. et al., 2017). With the aid of good solubility, similar two-dimensional geometry, the high theoretical capacity of MnO_2_, and the good conductivity of Ti_3_C_2_, the composite electrode exhibited good capacitance performance with a high mass-specific capacitance of 305 F/g at a current density of 1 A/g. The fabricated symmetrical flexible supercapacitor device exhibited a maximum energy density of 8.3 Wh/kg, and the power density could reach 2,376 W/kg (Figures 2E,F). Characterizations and deeper understanding of the interaction between the stacked hierarchical structure and electrochemical performance revealed that the layered stacking structure is more conducive to improve the electrochemical performance of the electrode compared with traditional capacitors.

Forming a heterostructure or core-shell structures has been adopted as an efficient strategy for offering a large surface area for more Faradaic reaction sites and high conductivity to accelerate the charge transfer and therefore to improve the electrochemical performances of nanocomposites. For instance, a ZnCo_2_O_4_-MnO_2_ heterostructure on Ni foam was shown to be an active electrode material with a porous nanostructure, providing a considerably large electroactive area, and exhibited ideal capacitive behavior, with a maximum Cs of 2,057 F/g at a current density of 1 A/g and cycling stability of 96.5% after 5,000 cycles (Kumbhar and Kim, 2018) (Figures 2G–I). Unique core-shell arrays of CoFe_2_O_4_@MnO_2_ on nickel foam were also studied as an electrode material for a supercapacitor. Compared to the individual CoFe_2_O_4_ and MnO_2_ nanosheets, the composite electrode exhibited much higher specific area capacitance of 3.59 F/cm^2^ (≈1,995 F/g) at a current density of 2 mA/cm^2^ and a smaller semicircle in EIS, indicating faster ion insertion/extraction during electrochemical reactions, which can all be attributed to the hierarchical core-shell nanostructure. The asymmetric supercapacitor assembled using a CoFe_2_O_4_@MnO_2_ electrode had maximum energy density and maximum power density of 37 Wh/kg and 4,800 W/kg, respectively, with long-term cycling stability (91.4% retention after 2,250 cycles) (Gao et al., 2017).

**Figure 3:**
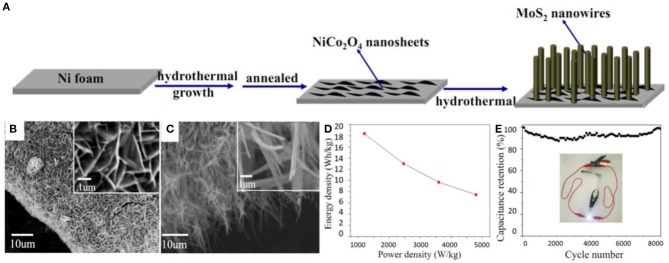
**(A)** Schematic illustration of the synthesis procedure of a MoS_2_ nanowires/NiCo_2_O_4_ nanosheets composite. **(B)** SEM images of the NiCo_2_O_4_ nanosheets supported on Ni foam. **(C)** SEM images of the composite supported on Ni foam. **(D)** Ragone plot and **(E)** cycling performance of the composite (Wen et al., 2018).

Besides, mixed dimensional hierarchical structures have also been reported. For instance, a hybrid of NiCo_2_O_4_-MoS_2_ consisting of 2D NiCo_2_O_4_ nanosheets and 1D MoS_2_ nanowires were grown on a 3D Ni foam network, forming mixed-dimensional hierarchical structures, as shown in Figures 3A–C. During the growth process, NiCo_2_O_4_ nanosheets intertwined on the Ni surface, providing sites for MoS_2_ nanowires, while a higher concentration of S^2−^ lead to thinner nanowires. These features enable a high specific surface area and a shortened diffusion path for ions and electrons, which lead to the enhancement of the electron/ion transfer rate in the electrodes (Wen et al., 2018). The assembled supercapacitor device delivered a maximum energy density of 18.4 Wh/kg and a power density of 1200.2 W/kg with excellent stability (specific capacitance retention of 98.2% after 8,000 cycles) (Figures 3D,E).

## TMOs/TMHs/Carbon-Based Hierarchical Structures

Graphene, described as the “famous star” among carbon materials (Gong et al., 2016), it is an excellent two-dimensional scaffold upon which to construct composite materials with 2D TMOs/TMHs because its large surface area and the huge amount of cross-linking of the conjugated π-bond structure endow it with superior conductivity (in-plane carrier mobility up to 200,000 cm^2^v^−1^s^−1^) and physical structure stability (high mechanical strength and flexibility) (Novoselov et al., 2012). The electronic conduction and mechanical stability of the hierarchical structure formed are improved by the synergistic effect of the two components (Stankovich et al., 2006). Hence, a supercapacitor with excellent performance is expected due to the improved electrochemical reaction rate and cycle stability.

**Figure 4:**
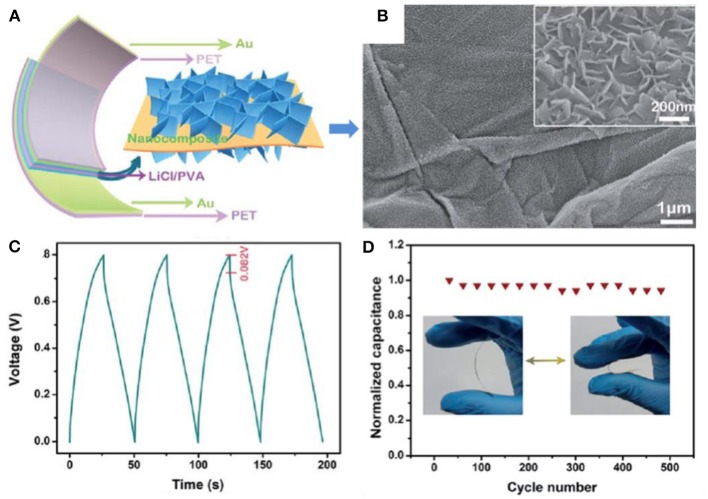
**(A)** Schematic illustration of the flexible ASSTFS. **(B)** SEM images of the as-obtained V_2_O_5_ nanosheet/graphene nanocomposite. **(C)** The first four cycles of a galvanostatic charge–discharge curve of the as-fabricated ASSTFS at a current density of 2.5 A/m^2^. **(D)** Long-term cycling stability investigation of the ASSTFS after repeated bending/extension for 500 cycles (Bao et al., 2014).

At present, a large amount of progress has been made in the preparation of high-performance energy storage materials by combining TMOs/TMHs with graphene. The application of pseudocapacitive V_2_O_5_ nanosheets and graphene in all-solid-state flexible thin-film supercapacitors (ASSTFSs) has been reported (Bao et al., 2014). By rationally integrating the two components, electron transfer was accelerated, and diffusion paths were shortened, leading to strong electrochemical performance, with a capacitance of 11,718 μF/cm^2^ at 0.2 A/m^2^, an energy density of 1.13 μW h/cm^2^ at a power density of 10.0 μW/cm^2^, and excellent long-term cycling stability for 2,000 charge–discharge cycles (Figure 4). The superior electrochemical performance thoroughly demonstrated the merit of a hierarchical architecture combining pseudocapacitive V_2_O_5_ nanosheets and graphene.

**Figure 5:**
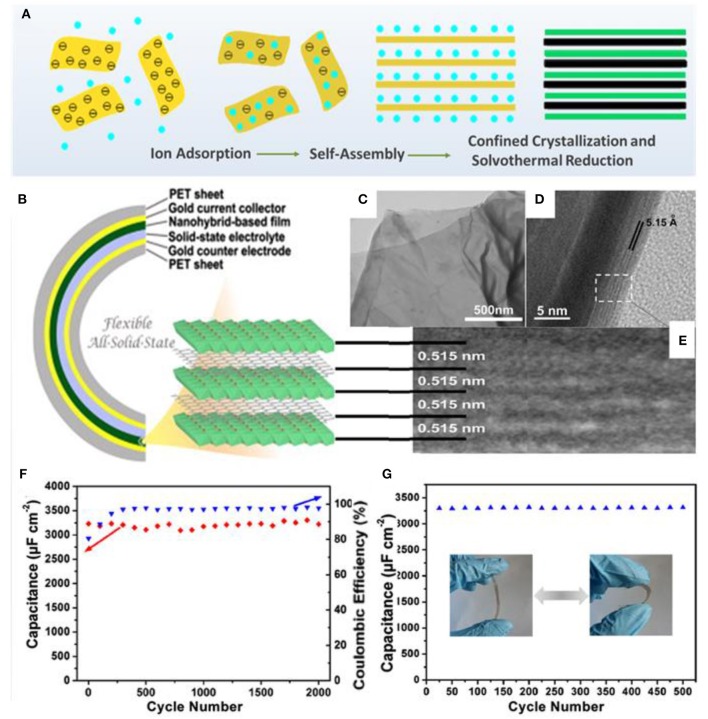
**(A)** Schematic illustration of the layer-by-layer formation mechanism of β-Ni(OH)_2_/graphene nanohybrids. **(B)** Schematic illustration of the flexible all-solid-state thin-film supercapacitor with pseudocapacitive β-Ni(OH)_2_/graphene nanohybrids as active materials. **(C)** TEM image of the as-prepared nanohybrids, confirming the nanosheet morphology. **(D)** Cross-sectional HR-TEM images and **(E)** enlarged view of the HR-TEM image of the curled fringe of the nanohybrid sheet. **(F)** Long-term cycling stability of the flexible ASSTFS based on the nanohybrids (98.2% for the 2,000th cycle). **(G)** Cycling stability of the flexible ASSTFS measured after repeated bending/extension deformation (Xie et al., 2013).

The Xie, group for the first time, fabricated a flexible all-solid-state thin-film pseudocapacitor using β-Ni(OH)_2_/graphene hybrid nanosheets as electrode materials via a layer-by-layer method (Xie et al., 2013). The assembly process of the β-Ni(OH)_2_/graphene hybrid includes electrostatic interaction between GO and Ni^2+^, interlayer Ni^2+^ diffusion, and confined (NiOH)_2_ crystallization, as well as simultaneous reduction of graphene (Figures 5A–E). The combination of highly conductive graphene and pseudocapacitive β-Ni(OH)_2_ guarantees high specific capacitance and excellent stability for this novel energy storage material. The fabricated all-solid-state thin-film pseudocapacitor exhibits a high volumetric specific capacitance of 660.8 F/cm and good cycling ability, as well as excellent flexibility with negligible degradation after 200 bending cycles (Figures 5F,G). Meanwhile, the ultrathin configuration of the hybrid nanosheets endows superior mechanical properties for the as-fabricated device, which can be regarded as a feasible energy supply for the exploitation of flexible electronics.

**Figure 6:**
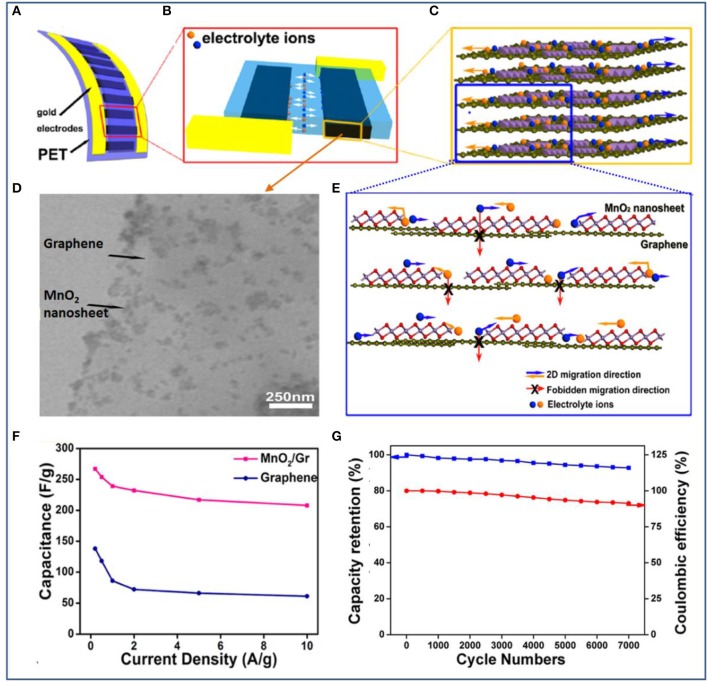
**(A)** Schematic illustration of an ultraflexible planar supercapacitor constructed with a hybrid film as the working electrode, a current collector, and a gel electrolyte on a plastic polyethylene terephthalate (PET) substrate. **(B)** The planar supercapacitor unit, showing that the 2D hybrid thin film functions as two symmetric working electrodes. **(C)** The hybrid thin film was formed by stacking layers of chemically integrated quasi-2D δ-MnO_2_ nanosheets and graphene sheets. **(D)** TEM image of the 2D hybrid structure with δ-MnO_2_ nanosheets integrated on graphene surfaces. **(E)** Schematic description of the 2D planar ion transport favored within the 2D δ-MnO_2_/graphene hybrid structures. **(F)** Comparison of specific capacitance values for the supercapacitors based on hybrids and based on graphene. **(G)** Capacitance retention (blue) and Coulombic efficiency (red) of the planar supercapacitor device based on hybrids over 7,000 charge/discharge cycles (Peng et al., 2013).

Following the all-solid-state planar configuration, the Xie group developed a quasi-2D ultrathin MnO_2_/graphene hybrid for fabricating a novel and high-performance planar supercapacitor (Peng et al., 2013). To make the best of the designed planar structure, a vacuum filtration method is adopted to produce films with controllable thickness and transferability. These hybrid 2D δ-MnO_2_/graphene thin films can be transferred onto a range of substrates such as PET, quartz, glass, and silicon wafer. By filling with a gel electrolyte of PVA/H_3_PO_4_, an all-solid-state planar supercapacitor is fabricated (Figures 6A,B). Owing to the planar peculiarity of both graphene and δ-MnO_2_ nanosheets, more electrochemically active surfaces for absorption/desorption of electrolyte ions were introduced, and charge transport was accelerated at the hybridized interlayer during the charging and discharging processes (Figures 6C–E). The device exhibited high specific capacitances of 267 F/g at a current density of 0.2 A/g and 208 F/g at 10 A/g and excellent rate capability and cycling stability (capacitance retention of 92% after 7,000 cycles) (Figures 6F,G).

In addition to these pioneering and representative research studies, numerous reports on the integration of TMOs/TMHs with graphene to prepare high-performance electrode materials for supercapacitors have revealed the general functions of the hierarchical structures (Wang G. et al., 2012; Mahmood et al., 2019; Nguyen and Montemor, 2019). The incorporation of graphene improves the conductivity of the pseudocapacitor electrode material and the constraints of the low specific capacitance of the graphene as a double-layer capacitor electrode material, and the advantages of the two are superimposed to achieve a substantial improvement in the capacitance performance, which is a feasible strategy for improving the electrochemical performance of the TMO/TMH electrode material.

## TMOs/TMHs/Conducting Polymer Hierarchical Structures

Conducting polymer has become an important electrode material for pseudocapacitors due to the advantages of large capacity, good conductivity, facile synthesis, and low cost (Kalaji et al., 1999). In the past few years, conducting polymers have attracted increasing attention due to their great potential in supercapacitors. However, the conductive polymer-based electrode still suffers the drawbacks of low stability and poor mechanical properties, which restrict its application in fabricating supercapacitor devices (Liu et al., 2019). In terms of countermeasures, an inorganic-organic composite combination can improve the mechanical properties, electrochemical properties, and stability. Organic-inorganic composite electrode materials with excellent performance can be prepared to improve the specific capacitance and cycle stability of the electrode materials of supercapacitors.

**Figure 7:**
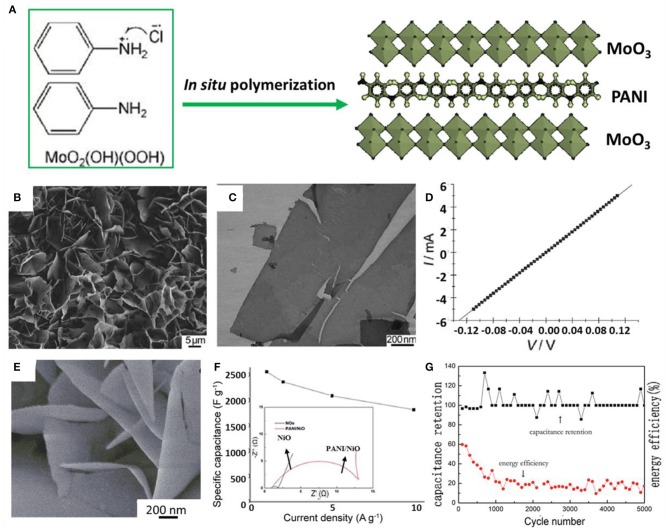
**(A)** Representation of the simultaneous reaction mechanism of hybrid PANI/MoO_3_ nanocomposites. **(B)** SEM images and **(C)** TEM images of the prepared hybrid PANI/MoO_3_ nanosheets. **(D)** I–V characteristics of the hybrid nanosheet (Zheng et al., 2011). **(E)** SEM images of the PANI/NiO electrode. **(F)** Specific capacity of the PANI/NiO electrode in different current densities and (inset) EIS curves. **(G)** Capacitance retention and energy efficiency curves of the PANI/NiO electrode produced in the best condition (Sun et al., 2016).

As a representative conducting polymer, polyaniline (PANI) has frequently been used to combine with TMOs/TMHs to fabricate hierarchical structural hybrids for supercapacitor materials. As reported earlier, MoO_3_/PANI nanocomposites have been synthesized by a simple *in-situ* synthesis method using molybdenum oxide precursor precipitation and aniline monomer as raw materials, as shown in Figures 7A–C (Zheng et al., 2011). The PANI polymer chains are assembled between oxide layers, which cause the nanocomposite to exhibit a flexible layered hierarchical structure. The electrical properties of metal oxides as active supercapacitor materials are greatly improved, which gives them excellent conductivity (Figure 7D). The outstanding performance was inferred to derive from the robust bonding through *in-situ* polymerization. Hence, nanocomposites prepared by *in-situ* reaction at the molecular level have significantly improved specific capacity and cycle stability, making them very suitable for the electrochemical energy storage application.

A PANI-NiO composite on nickel foam that was used as a supercapacitor electrode and was fabricated via a binder-free *in-situ* approach showed high specific capacitance (2,565 F/g at a current density of 1 A/g) and excellent cycling stability (with a high retention of 100% for almost 5,000 cycles) (Sun et al., 2016). The flower-like hierarchical structures offer a structural benefit enabling high levels of redox (Figures 7E–G). Detailed studies revealed that the Ni foam was the current collector and Ni source for *in-situ* deposition of NiO, establishing a steadier bonding between collector and active material. PANI that was deposited directly onto the electrode supported the structure and prevented the functional space structure from being destroyed and collapsing during the charging–discharging process. The resultant impressive cycling stability shows considerable potential for commonly used energy storage devices with long service lives.

**Figure 8:**
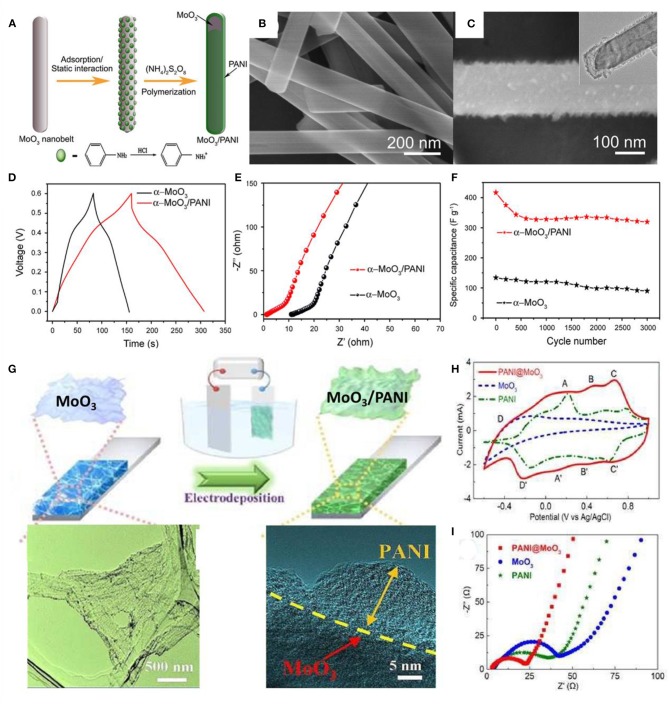
**(A)** Schematic illustration of the formation of the MoO_3_/PANI coaxial heterostructure nanobelts. **(B)** SEM images of the original α-MoO_3_ nanobelts. **(C)** SEM images and (inset) TEM images of the as-synthesized MoO_3_/PANI coaxial heterostructure nanobelts. **(D)** A comparison of the galvanostatic charge–discharge curves of the two comparative materials at a current density of 2 A/g. **(E)** EIS spectra comparison and **(F)** cycling performance at a scan rate of 50 mV/s of the two comparative materials (Jiang et al., 2014). **(G)** Schematic illustration for the synthesis process of 3D MoO_3_/PANI hybrid nanosheet network film. **(H)** CV curves of MoO_3_, PANI, and 3D MoO_3_/PANI hybrid nanosheet network films in the potential range from −0.6 to 1 V at a scanning rate of 50 mV/s. **(I)** Nyquist plots of the MoO_3_, PANI, and 3D MoO_3_/PANI hybrid nanosheet network films (Zhang et al., 2017).

Post-functionalization of TMOs/TMHs with PANI through polymerization provides the hybrid core-shell or sandwich hierarchical configurations. For example, adsorption of benzene amine on MoO_3_ nanobelts and further polymerization lead to the formation of coaxial heterostructure nanobelts of MoO_3_/PANI, which exhibited lower electric resistance than MoO_3_ nanobelts and pure PANI (Figures 8A–F). The supercapacitors achieved high specific capacitances of 714 F/g at a scan rate of I mV/s and 632 F/g at a current density of 1 A/g (Jiang et al., 2014).

In order to overcome the low stability of conducting polymers as electrode material, a porous structure is usually introduced to guard against swelling and shrinking. In the case of a MoO_3_/PANI hybrid (Zhang et al., 2017), 3D MoO_3_ networks are formed through a freeze-drying process, in which the ice acts as a self-sacrificial template, and the different nanosheets are connected by van der Waals and hydrogen bonds. The final 3D MoO_3_/PANI hybrid networks were achieved by further electropolymerization of PANI onto the MoO_3_ surface (Figure 8G). Electrochemical impedance characteristics (EIS) measurements demonstrated good electrical conductivity and ion diffusion behavior (Figures 8H,I). The intrinsic structural advantages and the synergetic interaction of the 3D networks make such a hierarchical configuration a promising structure for supercapacitor materials.

It can be seen from the above work that preparing inorganic-organic composite electrode materials by combining 2D TMOs/TMHs with conductive polymers can effectively improve the cycling, mechanical properties, and specific capacitance of the electrode, which can be used as an effective and feasible scheme for improving the electrochemical performance of the electrode of a supercapacitor.

## Summary and Perspectives

A 2D TMOs/TMHs-based hierarchical structure combines the advantages of planar conductivity and a large specific surface area, making it an ideal candidate for the assembly of high-performance supercapacitors. To this end, finding new configurations with a hierarchical structure will guide the fabrication of supercapacitor materials with further-improved electrochemical performances. Besides TMOs/TMHs hierarchical structures, the integration of TMOs/TMHs with graphene or conductive polymer to form hierarchical structures has directed the recent steps forward in supercapacitor materials. In TMOs/TMHs-based hierarchical structures, graphene and conductive polymer are the key components for constructing flexible supercapacitors. By optimizing the TMOs/TMHs nanosheet orientation and stacking, electrons/ion transport channels were created, and performance was enhanced in the hierarchical energy storage devices. In summary, hierarchical structures form the prospective blueprint for fabricating high-performance supercapacitors by regulating morphologies and electronic properties.

Although great efforts have been put into supercapacitor materials in terms of the development of novel materials, synthetic technology, and structure engineering, a few problems remain at present. For instance, in TMOs/TMHs/graphene hierarchical structures, the weak interaction forces between capacitive TMOs/TMHs and graphene at the heterostructure interface have been an impediment to improving electrochemical performance. However, very recently, graphene was demonstrated to eliminate the boundary effect and enable electron behavior of the hybrid comparable with that of a single crystal without grain boundary. It is believed that this discovery may inspire fresh research into hierarchical structures using graphene as a component. Given the advantages of conductive polymer, rational design and synthesis of new types of conductive polymers with higher conductivity and stability are highly desirable, as this would pave the way for next-generation supercapacitors with superior flexibility and performance.

Besides assembling hierarchical structures for improvement of supercapacitor performance, attention could be paid to developing new 2D electroactive materials, such as the newly emerging 2D transition metal carbides/nitrides (MXenes), which possess extremely high intrinsic electronic/ionic conductivity. High intercalation capacitance (~1,000 F cm^−3^) in aqueous electrolytes make these promising electrode materials for the future. Meanwhile, supercapacitor performance is also dependent on the voltage window, which is strictly limited by the electrolyte. Thus, a possible way forward is to increase the working voltage by using green electrolytes with a high electronic conductivity and voltage window, such as ionic liquid electrolytes.

## Author Contributions

All authors listed have made a substantial, direct and intellectual contribution to the work, and approved it for publication.

## Conflict of Interest

The authors declare that the research was conducted in the absence of any commercial or financial relationships that could be construed as a potential conflict of interest.
